# Proposal for Classifying the Emetogenicity of Oral Anticancer Agents with a Focus on PARP Inhibitors: A Prospective, Observational, Multicenter Study (JASCC-CINV 2002)

**DOI:** 10.7150/jca.91675

**Published:** 2024-01-21

**Authors:** Senri Yamamoto, Masami Tsuchiya, Hirotoshi Iihara, Yoh Hayasaki, Kyoko Hori, Yasuo Kumakura, Daichi Watanabe, Hideki Sakai, Satoshi Nakagawa, Akiko Kudoh, Hajime Oishi, Nobuhiro Kado, Makiko Go, Kota Mashima, Takashi Uchida, Moeka Yasue, Akimitsu Maeda, Kimihiro Nishino, Koji Matsumoto, Shinya Sato, Yutaka Ueda, Kensuke Tomio, Katsuhisa Hayashi, Motoki Takenaka, Masahiko Mori, Hiroaki Kajiyama, Yoshimasa Bomoto, Shiro Suzuki, Takuma Ishihara, Akio Suzuki, Masakazu Abe

**Affiliations:** 1 Department of Pharmacy, Gifu University Hospital, 1-1 Yanagido, Gifu, Gifu, 501-1194, Japan.; 2 Department of Pharmacy, Miyagi Cancer Center, 47-1 Nodayama, Medeshimashiote, Natori, Miyagi, 981-1293, Japan.; 3 Patient Safety Division, Gifu University Hospital, 1-1 Yanagido, Gifu, Gifu, 501-1194, Japan.; 4 Laboratory of Community Pharmaceutical Practice and Science, Gifu Pharmaceutical University, 1-25-4 Daigakunishi, Gifu, Gifu, 501-1196, Japan.; 5 Department of Obstetrics and Gynecology, Gifu University Graduate School of Medicine, 1-1 Yanagido, Gifu, Gifu, 501-1194, Japan.; 6 Department of Pharmacy, Aichi Cancer Center Hospital, 1-1 Kanokoden, Chikusa-ku, Nagoya, Aichi, 464-8681, Japan.; 7 Department of Hospital Pharmacy, Nagoya University Hospital, 65 Tsurumai-cho, Showa-ku, Nagoya Aichi, 466-8560, Japan.; 8 Innovative and Clinical Research Promotion Center, Gifu University Hospital, 1-1 Yanagido, Gifu, Gifu, 501-1194, Japan.; 9 Medical Oncology Division, Hyogo Cancer Center, 13-70, Kitaoji-cho, Akashi, Hyogo, 673-8558, Japan.; 10 Department of Obstetrics and Gynecology, Osaka University Graduate School of Medicine, 2-2 Yamadaoka, Suita Osaka, 565-0871, Japan.; 11 Department of Obstetrics and Gynecology, Tottori University Faculty of Medicine, 36-1 Nishi-cho, Yonago, Tottori, 683-8504, Japan.; 12 Department of Obstetrics and Gynecology, Center Hospital of the National Center for Global Health and Medicine 1-21-1 Toyama, Shinjuku-ku, Tokyo, 162-8655, Japan.; 13 Department of Gynecology, Shizuoka Cancer Center 1007 Shimonagakubo, Nagaizumi-cho, Sunto-gun, Shizuoka, 411-8777, Japan.; 14 Department of Pharmacy, Ogaki Municipal Hospital, 4-86 Minaminokawa-cho, Ogaki Gifu, 503-0896, Japan.; 15 Department of Pharmacy, Fukuoka University Hospital, 7-45-1 Nanakuma, Jounanku, Fukuokashi, Fukuoka, 814-0180, Japan.; 16 Department of Healthcare Administration, Nagoya University Graduate School of Medicine, 65 Tsurumai-cho, Showa-ku, Nagoya, 466-8550, Japan.; 17 Department of Gynecologic Oncology, Aichi Cancer Center Hospital, 1-1 Kanokoden, Chikusa-ku, Nagoya, Aichi, 464-8681, Japan.; 18 Department of Obstetrics and Gynecology, Nagoya University Graduate School of Medicine, 65 Tsuruma-cho, Showa-ku, Nagoya, Aichi, 466-8550, Japan; 19 Laboratory of Advanced Medical Pharmacy, Gifu Pharmaceutical University, 1-25-4 Daigakunishi, Gifu, 501-1196, Japan.; 20 Department of Obstetrics and Gynecology, Hamamatsu University School of Medicine, 1-20-1 Handayama, Higashi-ku, Hamamatsu City, Shizuoka, 431-3192, Japan.

**Keywords:** olaparib, niraparib, PARP inhibitor, nausea, vomiting, gynecologic cancer

## Abstract

**Background:** Olaparib and niraparib (poly adenosine diphosphate [ADP]-ribose polymerase [PARP] inhibitors) have significant antitumor action in patients with ovarian cancer. However, the incidence of nausea and vomiting among patients on these drugs in clinical trials is rather high. There are no guidelines on antiemetic treatment for nausea caused by oral anticancer agents. This study aimed to investigate the incidence of nausea and vomiting caused by PARP inhibitors and the actual situation of antiemetic therapy in patients with gynecologic cancer.

**Methods:** Patients with gynecologic cancer who were scheduled to receive PARP inhibitors were enrolled. Data on PARP inhibitor-induced nausea and vomiting were collected from patient diaries for 21 days. The primary endpoint was the incidence of vomiting during the 21 days after starting olaparib and niraparib.

**Results:** Overall, between January 2020 and March 2023, 134 patients were enrolled. Of the 129 patients who were evaluated, 28 (21.7%) received prophylactic antiemetics for 21 days, and 101 (78.3%) did not. The overall incidence of PARP inhibitor-induced vomiting was 16.3%. The incidence of vomiting in the group that did not receive antiemetic prophylaxis was 13.9%. On dividing the group that did not receive antiemetic prophylaxis into the olaparib and niraparib subgroups, the incidence of vomiting was found to be 18.6% for the olaparib group and 10.3% for the niraparib group.

**Conclusion:** The incidence of emesis without antiemetic prophylaxis among patients on olaparib and niraparib ranged from 10% to 30%. Therefore, olaparib and niraparib can be classified in the low emetogenic risk and prophylactic antiemetic therapy at the time of treatment initiation may be unnecessary.

## Introduction

Olaparib and niraparib are oral poly adenosine diphosphate [ADP]-ribose polymerase (PARP) inhibitors that have shown antitumor activity in patients with ovarian cancer and in multiple settings 1-7. Their combination with bevacizumab has also shown a high level of clinical activity in patients with ovarian cancer 8,9. In addition, olaparib is highly effective in patients with germline *BRCA* mutated-breast cancer 10, germline *BRCA*-mutated pancreatic cancer 11, and in prostate cancers with alterations in homologous recombination repair genes 12.

However, olaparib and niraparib have been associated with high rates of nausea and vomiting in clinical trials, with incidence rates of 75.9% and 37.4% in the SOLO2 trial 3, 77% and 40% in the SOLO1 trial 4, 53% and 22% in the PAOLA-1 trial 8, 57.4% and 22.3% in the PRIMA/ENGOT-OV26/GOG-3012 trial 5, 73.6% and 34.3% in the ENGOT-OV16/NOVA trial 6, 60.7% and 34.1% in the QUADRA trial 7, and 63% and 33.3% in the NSGO-AVANOVA2/ENGOT-ov24 trials for ovarian cancer 9; 58.0% and 29.8% in the OlympiAD trial for breast cancer 10; 45% and 20% in the POLO trial for pancreatic cancer 11; and 41% and 18% in the PROfound trial for prostate cancer, respectively.

The American Society of Clinical Oncology (ASCO) guidelines for chemotherapy-induced nausea and vomiting (CINV) classify olaparib as having minimal or low emetic risk and niraparib as having moderate or high emetic risk 13; the National Comprehensive Cancer Network (NCCN) classifies olaparib and niraparib as having moderate-to-high emetic risk 14; the Multinational Association of Supportive Care in Cancer (MASCC)/European Society for Medical Oncology classifies olaparib as having low emetic risk, with no mention of niraparib 15. The determination of emetogenic classification in these guidelines has been challenging due to the limited coverage of "common" toxicities, such as emesis during antineoplastic drug development and the unregulated use of prophylactic antiemetics during chemotherapy 15. With the exception of the NCCN guidelines, no other guidelines have provided antiemetic prophylaxis recommendations for oral anticancer agents. NCCN guidelines recommend 5-hydroxytryptamine-3 receptor antagonist (5-HT_3_RA) prophylaxis for drugs classified as moderate-to-high emetic-risk drugs such as olaparib and niraparib. This difference in the classification of emetic risk and recommendations for prophylactic antiemetic therapy among the guidelines can be attributed to the fact that the guidelines have evolved to cover parenteral injections. Moreover, there is no established method for assessing nausea and vomiting or for classifying the emetic risks of oral anticancer drugs. Uncontrolled nausea and vomiting induced by oral anticancer medications may directly lead to low or nonadherence to anticancer medications and affect treatment efficacy. Therefore, there is an unmet clinical need to control nausea and vomiting during oral chemotherapy.

This study aimed to investigate the incidence of nausea and vomiting caused by olaparib and niraparib and the current status of antiemetic therapy for preventing and treating nausea and vomiting in patients with gynecologic cancer.

## Materials and Methods

### Study Design

This prospective, observational, multicenter study was conducted in 13 Japanese hospitals (university hospitals, cancer centers, national centers, public hospitals, and private hospitals) in accordance with the Declaration of Helsinki and the Ethical Guidelines for Clinical Studies. The study was approved by the institutional review board of each participating center and was independently monitored by the alliance data center. Data collection and analysis were conducted by the Alliance Statistics and Data Management Center. Data quality was ensured by a review of the data performed by the Alliance Statistics and Data Management Center and by the principal investigator according to the policies of the Center. The study was registered with the University Hospital Medical Information Network (UMIN000039076).

### Patient Selection

Patients with ovarian cancer who were scheduled to receive olaparib or niraparib-containing anticancer chemotherapy for the first time were included in this study. Because niraparib was approved for marketing in Japan in November 2020, the protocol was revised to include patients with ovarian cancer who received niraparib in the trial. Other eligibility criteria included patients who could maintain an accurate diary, aged 20 years or older, and provision of written informed consent.

Patients were ineligible if they met any of the following criteria: started on olaparib or niraparib at a reduced dose; needed antiemetics at the time of enrolment; started taking opioids within 48 h prior to enrolment; had ascites requiring paracentesis; presence of symptomatic brain metastases and cancerous meningitis; presence of gastrointestinal obstruction; underwent abdominal or pelvic irradiation within 6 days prior to enrolment; no current use of medications with antiemetic activity such as neurokinin-1 receptor antagonist (NK_1_RA), 5-HT_3_RA, corticosteroids, dopamine antagonists, phenothiazine tranquilizers, selective serotonin reuptake inhibitors, serotonin and norepinephrine reuptake inhibitors, serotonin-dopamine antagonists, multi-acting receptor targeted antipsychotics, or benzodiazepines; and patients judged to be inappropriate for the study by the investigator.

### Assessment Procedures

All relevant demographic characteristics and medical data were recorded during the pre-study period. Data were collected from the patient diaries. Patients filled out a diary daily from the start of therapy with PARP inhibitors for 21 days. They reported decreased appetite (none, mild, moderate, and severe), presence of nausea (none, mild, moderate, and severe), vomiting (none, 1-2 times, 3-5 times, 6 times, or more), use of rescue medication (none, 1 time, 2 times, and 3 times, or more), number of defecations, and stool characteristics based on the Bristol stool form scale daily 16. In addition, the following items of the Patient Reported Outcomes version of the Common Terminology Criteria for Adverse Events (PRO-CTCAE), version 1.0, were reported every 7 days: nausea, vomiting, decreased appetite, taste changes, fatigue, constipation, diarrhea, and insomnia. Weight was recorded every 7 days. Assessments were performed before administering PARP inhibitors to collect baseline data.

Patient satisfaction in the absence of nausea or vomiting was measured every 7 days using a 7-point scale (very satisfied, satisfied, somewhat satisfied, somewhat satisfied, somewhat dissatisfied, dissatisfied, and very dissatisfied). After the overall assessment period, patient-reported study diaries were collected.

Study data were collected and managed using the Research Electronic Data Capture (REDCap) tool hosted by Gifu University Hospital 17,18. REDCap is a secure web-based software platform designed to support data capture for research studies, providing 1) an intuitive interface for validated data capture, 2) audit trails for tracking data manipulation and export procedures, 3) automated export procedures for seamless data downloads to common statistical packages, and 4) procedures for data integration and interoperability with external sources.

### Outcomes

The primary outcome was the incidence of vomiting during the first 21 days after starting PARP inhibitor therapy. In the primary analysis, vomiting was treated as time-to-event data for up to 21 days after the start of PARP inhibitor treatment until the first event. Patients for whom an event could not be identified within 21 days were censored based on the date of last confirmation.

The secondary outcomes were the incidence of nausea and significant nausea during the 21 days; days from initiation of PARP treatment to onset of vomiting, nausea, or significant nausea; percentage of patients receiving prophylactic antiemetic therapy; percentage of patients receiving antiemetic therapy and number of days receiving antiemetic therapy during the 21 days; reason and frequency of PARP dose reduction; body weight change; and patient satisfaction with the ability to live without the feeling of nausea and vomiting. The incidences of vomiting, nausea, and significant nausea were assessed according to the type and dose of antiemetic therapy administered and in patients taking concomitant CYP3A4 inhibitors. Adverse events were graded according to the Common Terminology Criteria for Adverse Events (CTCAE), version 5.0, and PRO-CTCAE, version 1.0.

Among patients receiving prophylactic antiemetics, the complete response (CR) rate was defined as no emetic episodes and no use of rescue medication during the 21 days. Complete control (CC) rate was defined as no emetic episodes, no rescue medication use, and no significant nausea during the 21 days. Significant nausea was defined as no more than “moderate” and “severe” categories. The total control (TC) rate was defined as no emetic episodes, no rescue medication use, and no nausea during the 21 days.

### Statistical Analysis

From the start of olaparib or niraparib use till day 21, approximately 30% of the patients without prophylactic antiemetics were expected to experience vomiting 19,20. A risk reduction of more than 20%, considered clinically meaningful, was expected with prophylactic antiemetics. The proportion of the use of prophylactic antiemetic therapy could be as low as 10% of the total sample. Therefore, assuming a 1:9 ratio of patients with and without antiemetic therapy, the number of cases required to detect a 20% difference in the antiemetic rate with a one-sided significance level of 2.5% and a power of 80% by the log-rank test was calculated to be 210 cases by Schoenfeld's method. Proportional hazard was assumed, and the hazard ratio was estimated as “log (antiemetic rate with antiemetic treatment)/log (antiemetic rate without antiemetic treatment).” A sample size of 234 patients was calculated, with an expected dropout rate of 10% after follow-up (with antiemetic therapy: 24 cases, without antiemetic therapy: 210 cases). Although the actual number of patients could not be enrolled as planned due to the coronavirus outbreak, as many patients as possible were enrolled.

Patient characteristics represented by continuous variables were described as median and interquartile range (IQR) and those represented by categorical variables, as count and proportion. The incidences of vomiting, nausea, and significant nausea were summarized according to the use of olaparib and niraparib in the presence or absence of prophylactic antiemetics. The number of days of onset of vomiting, nausea, significant nausea, anorexia, and weight fluctuations were summarized using the median and IQR. A Cox proportional hazards regression model was used to evaluate the effects of prophylactic antiemetics on vomiting and nausea. In addition to prophylactic antiemetics, the model also included age and previous experience with CINV as covariates. Patient satisfaction was summarized according to the frequency of each of the seven tiers. Each adverse event was summarized in terms of frequency by CTCAE grade. Statistical significance was set at a two-sided p-value <0.05. All analyses were performed using the R software version 4.3.1 (www.r-project.org).

### Ethical Considerations

This study was approved by the institutional review board of each participating institute. All patients provided written informed consent prior to study initiation.

## Results

### Study Patients and Antiemetic Treatment

**Figure 1:**
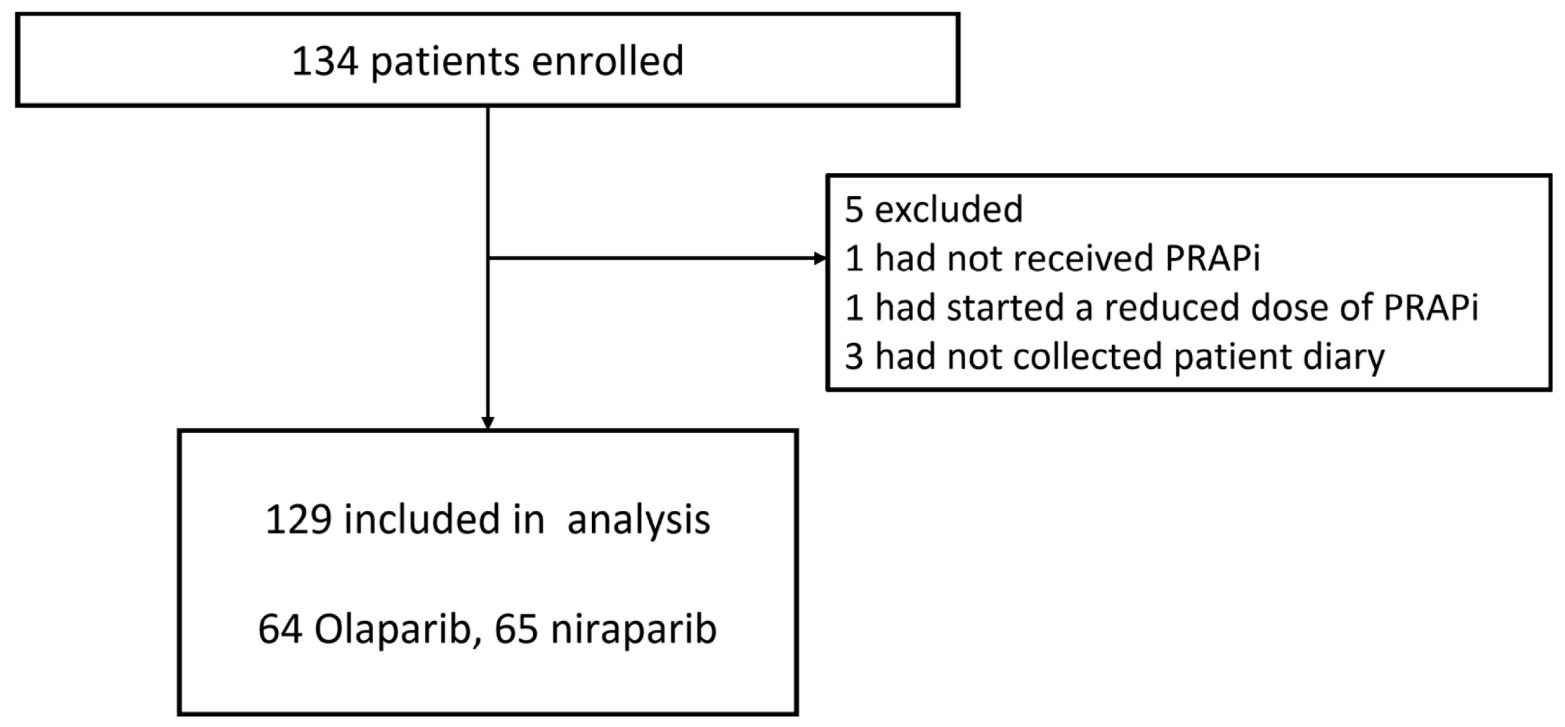
Trial profile.

A total of 134 patients were enrolled between January 2020 and March 2023 (Figure 1). Patients were expected to be enrolled over 2 years; however, the enrolment period was extended by 1 year due to the coronavirus disease 2019 (COVID-19) pandemic, which made it difficult for some participating centers to conduct clinical trials. Enrolment was stopped after 3 years due to the restrictions facing the healthcare system because of COVID-19. Therefore, although 234 patients were planned to be enrolled in this study, only 134 were enrolled. A total of 129 patients were included in the analysis, excluding one patient who did not receive PARP inhibitors, one who was started on a reduced dose of PARP inhibitor, and three patients whose patient diaries were not collected. The demographic data and patient characteristics are shown in Table 1. Of these patients, 28 (21.7%) were administered prophylactic antiemetics orally for 21 days during the observation period, and 101 (78.3%) did not receive any prophylaxis. No institution consistently administered prophylactic antiemetics. Prophylactic antiemetic administration was decided by the attending physician, based on the patient's condition, including experience with nausea and vomiting from previous therapy. None of the patients were treated with concomitant CYP3A4 inhibitors. The dose was reduced in five (3.9%) patients during the observation period due to hematologic toxicity.

The following antiemetics were administered in the group receiving prophylactic doses: prochlorperazine at 10 mg/day for seven patients and 15 mg/day for nine patients and metoclopramide at 5 mg/day for one patient and 15 mg/day in 11 patients. Five patients (17.9%) in the prophylaxis group and nine (8.9%) in the no-prophylaxis group received additional antiemetic therapy after initiation of PARP inhibitors; of the nine, three patients received daily oral medication to supplement antiemetic therapy. The durations of administration were 7, 9, and 15 days over the 21-day period.

### Incidence of Nausea and Vomiting and Body Weight Change

The incidence of nausea and vomiting after PARP inhibitor administration is shown in Table 2. The overall incidence of PARP inhibitor-induced vomiting, the primary outcome, was 16.3% (21 out of 129 patients). The overall incidence of vomiting was 25.0% (seven patients) in the prophylaxis group and 13.9% (14 patients) in the no-prophylaxis group. Vomiting with and without antiemetic prophylaxis was observed in six (28.6%) and eight (18.6%) patients on olaparib, respectively, and in one (14.3%) and six (10.3%) patients on niraparib, respectively.

**Figure 2:**
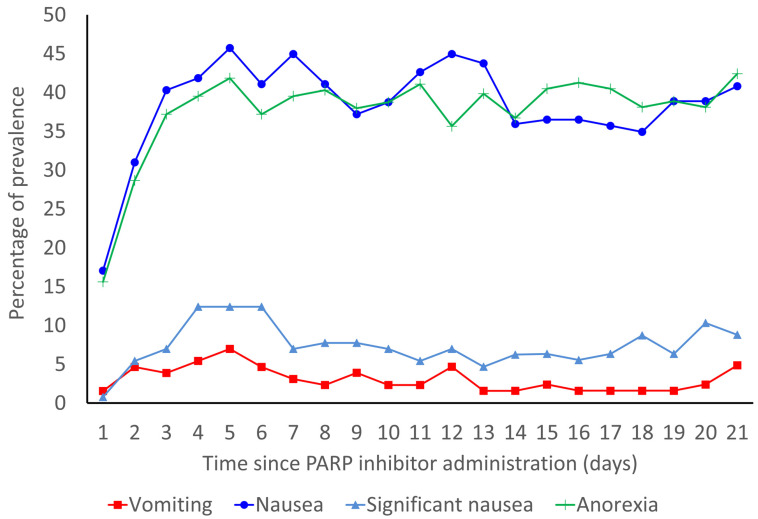
Prevalence rates of vomiting, nausea, significant nausea, and anorexia for 21 days from the start of PARP inhibitors.

The prevalence rates of vomiting, nausea, significant nausea, and anorexia are shown in Figure 2. In the overall population, the median durations of onset of vomiting, nausea, significant nausea, and anorexia were 4.5 (IQR: 2.0-9.8), 3.0 (IQR: 2.0-5.0), 5.0 (IQR: 3.0-14.0), and 3.0 (IQR: 2.0-6.0) days, respectively. Without antiemetic prophylaxis, the median durations of onset of vomiting, nausea, significant nausea, and anorexia were 4.5 (IQR: 2.25-8.25), 3.0 (IQR: 1.25-5.00), 5.0 (IQR: 3.75-13.25), and 3.0 (IQR: 2.00-6.25) days, respectively.

Weight fluctuations during the treatment period did not lead to a marked difference from the baseline weight: median, -0.1 kg (IQR: -1.0-0.55 kg) on day 7; median, -0.1 kg (IQR: -1.0-0.60 kg) on day 14; and median, -0.1 kg (IQR: -0.9-0.60 kg) on day 21.

### Association between Prophylactic Administration of Antiemetics and PARP Inhibitor-induced Nausea and Vomiting

The CR, CC, and TC rates were 64.3%, 46.4%, and 25.0%, respectively, over the 21-day period among patients who received prophylactic antiemetic therapy. On dividing these patients into the olaparib and niraparib subgroups, the CR, CC, and TC rates were found to be 61.9%, 38.1%, and 23.8%, respectively, for the olaparib group and 71.4%, 71.4%, and 28.6%, respectively, for the niraparib group.

Results of Cox proportional regression analysis of vomiting (hazard ratio [HR]: 1.899; 95% confidence interval [CI]: 0.766-4.708; *P* = 0.166), nausea (HR: 1.088; 95% CI: 0.666-1.778; *P* = 0.737), and significant nausea (HR: 1.206; 95% CI: 0.592-2.457; *P* = 0.606) are shown in Table 3. Antiemetic prophylaxis was not associated with a reduction in the incidences of vomiting, nausea, or significant nausea.

### Patient Satisfaction

During the 21-day observation period, overall, 85.9% (110/128) of the patients in the overall study group, 88.9% (24/27) of those who received antiemetic prophylaxis, and 86.9% (86/99) of those who did not were "somewhat satisfied" or feeling better with their ability to live without the feeling of nausea and vomiting.

### Adverse Events

**Figure 3:**
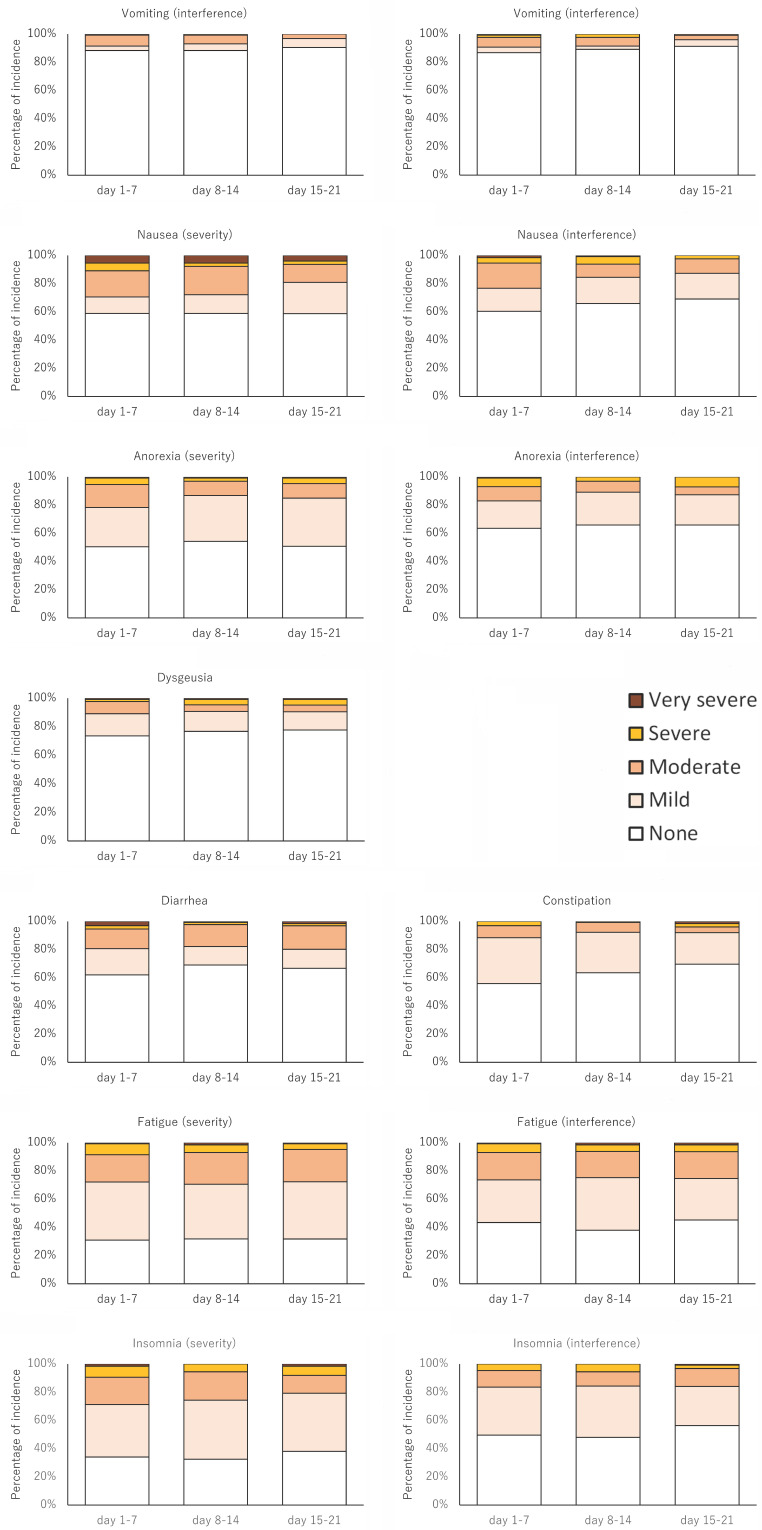
Changes over time in PRO-CTCAE.

The major adverse events are summarized in Table 4 and Figure 3. The percentages of severe or very severe adverse events were higher according to the PRO-CTCAE than according to the CTCAE.

## Discussion

To the best of our knowledge, this prospective observational study is the first to evaluate the emetogenicity of PARP inhibitors using patient diaries, with a focus on the occurrence of nausea and vomiting. In the present study, the incidence of vomiting was 13.9% in the group that did not receive antiemetic prophylaxis, 18.6% in the olaparib group, and 10.3% in the niraparib group. According to the ASCO and NCCN guidelines, the assessment of the emetogenic potential of oral chemotherapy agents is defined as "the proportion of patients who experience emesis in the absence of effective antiemetic prophylaxis.” This criterion is used to classify the emetogenic potential of drugs as moderate or high (≥30%) and minimal or low (<30%) 13,14. The MASCC guidelines classify oral chemotherapy agents as having high (>90%), moderate (30-90%), low (10-30%), and minimal (<10%) emetogenic potential similar to intravenous chemotherapy agents 15. Based on the results of the current study, the emetogenicity of olaparib and niraparib can be classified as minimal or low as per the ASCO and NCCN criteria and low as per the MASCC criteria. Olaparib has a terminal plasma half-life of approximately 15 h, whereas niraparib has a half-life of 36 h. Thus, the 21-day observation period was considered appropriate for evaluating nausea and vomiting, as both drugs are in a steady state 19,20.^19,20^ Reflecting the time it takes for these plasma concentrations to reach a steady state, the median time to onset of vomiting was 4.5 days, with a peak of 5 days.

The emetogenicity of intravenous chemotherapeutic agents is determined according to the percentage of patients who experience acute emesis in the absence of antiemetic prophylaxis 13-15. Considering the timing of emesis onset in the present study, we propose that the classification of emetogenicity of oral molecularly targeted agents, such as PARP inhibitors, which are administered daily with no rest periods, should be based on the onset of emesis during the observation period from the start of medication to 24 h after each drug reaches a steady state in the absence of antiemetic therapy. We expect these data to be obtained during the development of medicines.

In the current study, there was no difference in the incidence of vomiting, nausea, or significant nausea between the patients who received prophylactic antiemetic therapy and those who did not. As this was an observational study, patients who received antiemetic prophylaxis may have been those for whom the physician predicted the occurrence of nausea and vomiting. However, the Cox proportional regression analysis, adjusted for age and previous experience of CINV, the most relevant patient risk factors in this study, found no effect of prophylactic antiemetic administration 21. Therefore, with PARP inhibitors, as needed (pro re nata: PRN), initial antiemetic dosing is more appropriate than antiemetic prophylaxis, which is started before anticancer therapy and continued daily. Considering adverse events resulting from antiemetic prophylaxis, such as extrapyramidal symptoms caused by dopamine D2 receptor antagonists, this is acceptable given the high rate of “somewhat satisfied” or feeling better (86.9%) in our sample.

The prophylactic antiemetics used in this study were dopamine D_2_ receptor antagonists. Until the late 1970s, dopamine D_2_ receptor antagonists formed the basis of antiemetic therapy; however, the focus of antiemetic therapy shifted to 5HT_3_RA, NK_1_RA, and olanzapine, which have shown better efficacy as intravenous chemotherapy agents 22,23. Based on these findings and the results of this study that showed poor efficacy of dopamine D_2_ receptor antagonists, 5HT_3_RA and olanzapine, which are easy to administer orally, would be good candidate antiemetic agents for use PRN. In addition, among the adverse events observed in this study, the incidences of fatigue and insomnia were high. Fatigue and insomnia are patient-related characteristics that may increase the risk of chemotherapy-induced emesis; hence, measures other than antiemetic therapy should be considered 22.

This study has some limitations. First, this was an observation study. Therefore, even with appropriate inclusion and exclusion criteria, the occurrence of nausea and vomiting may be influenced by various factors, including unmeasured factors. Second, these results were obtained only in the Japanese population. However, because there are few racial differences in CINV, these results can be extrapolated to other patients worldwide. Third, although the indications for PARP inhibitors have been expanded to include a variety of diseases, the present study is the only one with results pertaining to female patients with gynecologic cancer. To our knowledge, this is the first study on PARP inhibitor-induced nausea and vomiting in a real-world population of young women at high risk of nausea and vomiting. Finally, the number of enrolled patients decreased to 134 owing to changes in the medical environment caused by COVID-19. The number of enrolled cases was calculated by assuming that 10% of all patients who did not receive prophylactic antiemetic therapy would have a 30% incidence of emesis, and those who received prophylactic antiemetic therapy would have experienced a 20% improvement. In this study, 21.7% of the patients received prophylactic treatment, which is higher than the 10% expected at design; therefore, we believe enough patients were enrolled to evaluate the efficacy of prophylactic antiemetic treatment.

In conclusion, we found that olaparib and niraparib should be classified in the low emetogenic risk category because the incidence of emesis without antiemetic prophylaxis ranged from 10% to 30%. Therefore, prophylactic antiemetic therapy at the initiation of treatment may be unnecessary. Oral antiemetic drugs other than dopamine D_2_ receptor antagonists should be considered PRN, and prospective interventional studies should be conducted to evaluate the efficacy of 5HT_3_RA and olanzapine.

## Figures and Tables

**Table 1:** Patients' characteristics

Characteristic	N = 129	
	n	(%)
**Age**		
Median	62	
IQR	54-71	
**Chemotherapy regimen**		
Olaparib	42	(32.6%)
Olaparib+bevacizumab	22	(17.1%)
Niraparib	64	(49.6%)
Niraparib+pertuzumab+trastuzumab	1	(0.8%)
**Maintenance therapy**		
Yes	115	(89.1%)
No	14	(10.9%)
**Treatment lines before maintenance therapy**		
1	85	(73.9%)
2	24	(20.9%)
3	4	(3.5%)
4	1	(0.9%)
6	1	(0.9%)
**Treatment lines in recurrence (withoout maintenace)**		
1	5	(35.7%)
2	5	(35.7%)
3	2	(14.3%)
4	1	(7.1%)
5	1	(7.1%)
**ECOG Performance Status**		
0	120	(93.0%)
1	9	(7.0%)
**Prophylactic antiemetic administration**		
Yes	28	(21.7%)
No	108	(78.3%)
**Motion sickness**		
Yes	35	(27.1%)
No	93	(72.1%)
Unknown	1	(0.8%)
**Morning sickness**		
Yes	60	(46.5%)
No	49	(38.0%)
No experience of pregnancy	19	(14.7%)
Unknown	1	(0.8%)
**Habitual alcohol consumption**		
Yes	35	(27.1%)
No	93	(72.1%)
Unknown	1	(0.8%)
**Previous experience of nausea and vomiting with chemotherapy**		
Yes	63	(48.8%)
No	65	(50.4%)
Unknown	1	(0.8%)

Abbreviations, ECOG: Eastern Cooperative Oncology Group; IQR: interquartile range

**Table 2:** Incidence of vomiting, nausea, and significant nausea for 21 days after the start of PARP inhibitors

PARPi	Groups	n (%)					
		Vomiting		Nausea		Significant nausea	
Overall	All (N=129)	21	(16.3%)	92	(71.3%)	42	(32.6%)
	With antiemetic prophylaxis (N=28)	7	(25.0%)	21	(75.0%)	10	(35.7%)
	Without antiemetic prophylaxis (N=101)	14	(13.9%)	71	(70.3%)	32	(31.7%)
Olaparib	All (N=64)	14	(21.9%)	52	(81.3%)	26	(40.6%)
	With antiemetic prophylaxis (N=21)	6	(28.6%)	16	(76.2%)	10	(47.6%)
	Without antiemetic prophylaxis (N=43)	8	(18.6%)	36	(83.7%)	16	(37.2%)
Niraparib	All (N=65)	7	(10.8%)	40	(61.5%)	16	(24.6%)
	With antiemetic prophylaxis (N=7)	1	(14.3%)	5	(71.4%)	0	(0%)
	Without antiemetic prophylaxis (N=58)	6	(10.3%)	35	(60.3%)	16	(27.6%)

Abbreviations, PARPi: poly (adenosine diphosphate [ADP]-ribose) polymerase (PARP) inhibitor

**Table 3:** Cox proportional hazards regression analysis to evaluate the effect of prophylactic antiemetics on vomiting, nausea, and significant nausea

PARPi	Outcomes	Univariable analysis				Multivariable analysis*		
		HR	95% CI	*p*-Value		HR	95% CI	*p*-Value
Overall	Vomiting	1.894	0.764-4.692	0.168		1.899	0.766-4.708	0.166
	Nausea	1.058	0.650-1.722	0.819		1.088	0.666-1.778	0.737
	Significant nausea	1.163	0.572-2.367	0.676		1.206	0.592-2.457	0.606
Olaparib	Vomiting	1.548	0.537-4.463	0.418		1.519	0.526-4.391	0.440
	Nausea	0.850	0.472-1.533	0.590		0.806	0.446-1.457	0.475
	Significant nausea	1.318	0.598-2.907	0.494		1.367	0.616-3.036	0.442
Niraparib	Vomiting	1.313	0.158-10.909	0.801		1.624	0.192-13.732	0.656
	Nausea	0.953	0.373-2.434	0.920		1.055	0.404-2.758	0.912
	Significant nausea**	0.001	0.000-6.274×10^17^	0.773		0.001	0.000-5.067×10^17^	0.768

Abbreviations, HR: Hazard ratio; 95% CI: 95% confidence interval range *Covariates: age and previous experience of nausea and vomiting with chemotherapy **The observed wide confidence intervals for the hazard ratios are attributable to the no incidence of significant nausea among patients using prophylactic antiemetics.

**Table 4:** Adverse events

PARPi	Symptom Term		PRO-CTCAE n (%)						CTCAE n (%)				
			All	Mild	Moderate	Severe	Very severe		All	Grade 1	Grade 2	Grade 3	Grade 4
Overall	Vomiting	(severity)	23 (17.8)	10 (7.8)	12 (9.3)	0 (0.0)	1 (0.8)		21 (16.3)	18 (14.0)	3 (2.3)	0 (0.0)	0 (0.0)
		(interference)	23 (17.8)	7 (5.4)	11 (8.5)	4 (3.1)	1 (0.8)						
	Nausea	(severity)	69 (53.5)	23 (17.8)	29 (22.5)	5 (3.9)	12 (9.3)		80 (62.0)	54 (41.9)	25 (19.4)	1 (0.8)	0 (0.0)
		(interference)	63 (48.8)	26 (20.2)	25 (19.4)	10 (7.8)	2 (1.6)						
	Anorexia	(severity)	81 (62.8)	43 (33.3)	25 (19.4)	10 (7.8)	3 (2.3)		75 (58.1)	52 (40.3)	21 (16.3)	2 (1.6)	0 (0.0)
		(interference)	62 (48.1)	33 (25.6)	15 (11.6)	13 (10.1)	1 (0.8)						
	Dysgeusia		42 (32.6)	24 (18.6)	11 (8.5)	6 (4.7)	1 (0.8)		30 (23.3)	24 (18.6)	6 (4.7)	0 (0.0)	0 (0.0)
	Constipation		69 (53.5)	44 (34.1)	17 (13.2)	6 (4.7)	2 (1.6)		53 (41.1)	40 (31.0)	12 (9.3)	1 (0.8)	0 (0.0)
	Diarrhea		63 (48.8)	27 (20.9)	27 (20.9)	4 (3.1)	5 (3.9)		26 (20.2)	23 (17.8)	3 (2.3)	0 (0.0)	0 (0.0)
	Fatigue	(severity)	101 (78.3)	45 (34.9)	38 (29.5)	15 (11.6)	3 (2.3)		72 (55.8)	51 (39.5)	20 (15.5)	1 (0.8)	0 (0.0)
		(interference)	91 (70.5)	41 (31.8)	32 (24.8)	15 (11.6)	3 (2.3)						
	Insomnia	(severity)	97 (75.2)	51 (39.5)	28 (21.7)	15 (11.6)	3 (2.3)		59 (45.7)	53 (41.1)	6 (4.7)	0 (0.0)	0 (0.0)
		(interference)	78 (60.5)	46 (35.7)	22 (17.1)	9 (7.0)	1 (0.8)						
													
Olaparib	Vomiting	(severity)	15 (23.4)	4 (6.2)	10 (15.6)	0 (0)	1 (1.6)		14 (21.9)	11 (17.2)	3 (4.7)	0 (0)	0 (0)
		(interference)	15 (23.4)	4 (6.2)	7 (10.9)	3 (4.7)	1 (1.6)						
	Nausea	(severity)	45 (70.3)	14 (21.9)	19 (29.7)	4 (6.2)	8 (12.5)		48 (75.0)	29 (45.3)	18 (28.1)	1 (1.6)	0 (0)
		(interference)	41 (64.1)	15 (23.4)	15 (23.4)	9 (14.1)	2 (3.1)						
	Anorexia	(severity)	48 (75.0)	22 (34.4)	19 (29.7)	4 (6.2)	3 (4.7)		48 (75.0)	32 (50.0)	15 (23.4)	1 (1.6)	0 (0)
		(interference)	41 (64.1)	23 (35.9)	7 (10.9)	10 (15.6)	1 (1.6)						
	Dysgeusia		30 (46.9)	12 (18.8)	11 (17.2)	6 (9.4)	1 (1.6)		26 (40.6)	20 (31.2)	6 (9.4)	0 (0)	0 (0)
	Constipation		31 (48.4)	23 (35.9)	6 (9.4)	1 (1.6)	1 (1.6)		26 (40.6)	16 (25.0)	9 (14.1)	1 (1.6)	0 (0)
	Diarrhea		39 (60.9)	14 (21.9)	19 (29.7)	2 (3.1)	4 (6.2)		20 (31.2)	17 (26.6)	3 (4.7)	0 (0)	0 (0)
	Fatigue	(severity)	54 (84.4)	20 (31.2)	22 (34.4)	10 (15.6)	2 (3.1)		41 (64.1)	23 (35.9)	17 (26.6)	1 (1.6)	0 (0)
		(interference)	51 (79.7)	19 (29.7)	19 (29.7)	11 (17.2)	2 (3.1)						
	Insomnia	(severity)	49 (76.6)	29 (45.3)	14 (21.9)	5 (7.8)	1 (1.6)		31 (48.4)	27 (42.2)	4 (6.2)	0 (0)	0 (0)
		(interference)	41 (64.1)	26 (40.6)	12 (18.8)	3 (4.7)	0 (0)						
													
Niraparib	Vomiting	(severity)	8 (12.3)	6 (9.2)	2 (3.1)	0 (0)	0 (0)		7 (10.8)	7 (10.8)	0 (0)	0 (0)	0 (0)
		(interference)	8 (12.3)	3 (4.6)	4 (6.2)	1 (1.5)	0 (0)						
	Nausea	(severity)	24 (36.9)	9 (13.8)	10 (15.4)	1 (1.5)	4 (6.2)		32 (49.2)	25 (38.5)	7 (10.8)	0 (0)	0 (0)
		(interference)	22 (33.8)	11 (16.9)	10 (15.4)	1 (1.5)	0 (0)						
	Anorexia	(severity)	33 (50.8)	21 (32.3)	6 (9.2)	6 (9.2)	0 (0)		27 (41.5)	20 (30.8)	6 (9.2)	1 (1.5)	0 (0)
		(interference)	21 (32.3)	10 (15.4)	8 (12.3)	3 (4.6)	0 (0)						
	Dysgeusia		12 (18.5)	12 (18.5)	0 (0)	0 (0)	0 (0)		4 (6.2)	4 (6.2)	0 (0)	0 (0)	0 (0)
	Constipation		38 (58.5)	21 (32.3)	11 (16.9)	5 (7.7)	1 (1.5)		27 (41.5)	24 (36.9)	3 (4.6)	0 (0)	0 (0)
	Diarrhea		24 (36.9)	13 (20.0)	8 (12.3)	2 (3.1)	1 (1.5)		6 (9.2)	6 (9.2)	0 (0)	0 (0)	0 (0)
	Fatigue	(severity)	47 (72.3)	25 (38.5)	16 (24.6)	5 (7.7)	1 (1.5)		31 (47.7)	28 (43.1)	3 (4.6)	0 (0)	0 (0)
		(interference)	40 (61.5)	22 (33.8)	13 (20.0)	4 (6.2)	1 (1.5)						
	Insomnia	(severity)	48 (73.8)	22 (33.8)	14 (21.5)	10 (15.4)	2 (3.1)		28 (43.1)	26 (40.0)	2 (3.1)	0 (0)	0 (0)
		(interference)	37 (56.9)	20 (30.8)	10 (15.4)	6 (9.2)	1 (1.5)						

## Abbreviations

PARP: poly adenosine diphosphate [ADP]-ribose polymerase
ASCO: American Society of Clinical Oncology
CINV: chemotherapy-induced nausea and vomiting
NCCN: National Comprehensive Cancer Network
MASCC: Multinational Association of Supportive Care in Cancer
5-HT_3_RA: 5-hydroxytryptamine-3 receptor antagonist
NK_1_RA: neurokinin-1 receptor antagonist
PRO-CTCAE: Patient Reported Outcomes version of the Common Terminology Criteria for Adverse Events
REDCap: Research Electronic Data Capture
CC: complete control
CR: complete response
TC: total control
HR: hazard ratio
CI: confidence interval

## References

[1] Ledermann J, Harter P, Gourley C (2012). Olaparib maintenance therapy in platinum-sensitive relapsed ovarian cancer. N Engl J Med.

[2] Ledermann J, Harter P, Gourley C (2014). Olaparib maintenance therapy in patients with platinum-sensitive relapsed serous ovarian cancer: a preplanned retrospective analysis of outcomes by BRCA status in a randomised phase 2 trial. Lancet Oncol.

[3] Pujade-Lauraine E, Ledermann JA, Selle F (2017). Olaparib tablets as maintenance therapy in patients with platinum-sensitive, relapsed ovarian cancer and a BRCA1/2 mutation (SOLO2/ENGOT-Ov21): a double-blind, randomised, placebo-controlled, phase 3 trial. Lancet Oncol.

[4] Moore K, Colombo N, Scambia G (2018). Maintenance olaparib in patients with newly diagnosed advanced ovarian cancer. N Engl J Med.

[5] González-Martín A, Pothuri B, Vergote I (2019). Niraparib in patients with newly diagnosed advanced ovarian cancer. N Engl J Med.

[6] Mirza MR, Monk BJ, Herrstedt J (2016). Niraparib maintenance therapy in platinum-sensitive, recurrent ovarian cancer. N Engl J Med.

[7] Moore KN, Secord AA, Geller MA (2019). Niraparib monotherapy for late-line treatment of ovarian cancer (QUADRA): a multicentre, open-label, single-arm, phase 2 trial. Lancet Oncol.

[8] Ray-Coquard I, Pautier P, Pignata S (2019). Olaparib plus bevacizumab as first-line maintenance in ovarian cancer. N Engl J Med.

[9] Mirza MR, Åvall Lundqvist E, Birrer MJ (2019). Niraparib plus bevacizumab versus niraparib alone for platinum-sensitive recurrent ovarian cancer (NSGO-AVANOVA2/ENGOT-ov24): a randomised, phase 2, superiority trial. Lancet Oncol.

[10] Robson M, Im SA, Senkus E (2017). Olaparib for metastatic breast cancer in patients with a germline BRCA mutation. N Engl J Med.

[11] Golan T, Hammel P, Reni M (2019). Maintenance olaparib for germline BRCA-mutated metastatic pancreatic cancer. N Engl J Med.

[12] de Bono J, Mateo J, Fizazi K (2020). Olaparib for metastatic castration-resistant prostate cancer. N Engl J Med.

[13] Hesketh PJ, Kris MG, Basch E (2020). Antiemetics: ASCO Guideline update. J Clin Oncol.

[14] NCCN Clinical Practice Guidelines in oncology: antiemesis, version 1.2023.

[15] MASCC/ESMO Antiemetic Guideline 2016 with updates in 2019.

[16] O'Donnell LJ, Virjee J, Heaton KW (1990). Detection of pseudodiarrhoea by simple clinical assessment of intestinal transit rate. BMJ.

[17] Harris PA, Taylor R, Thielke R (2009). Research electronic data capture (REDCap)-a metadata-driven methodology and workflow process for providing translational research informatics support. J Biomed Inform.

[18] Harris PA, Taylor R, Minor BL (2019). The REDCap consortium: building an international community of software platform partners. J Biomed Inform.

[19] LYNPARZA® (olaparib) tablets, for oral use. NDA 208558/S-025.

[20] ZEJULA® (niraparib) capsules, for oral use. NDA. 208447/S-026.

[21] Dranitsaris G, Molassiotis A, Clemons M (2017). The development of a prediction tool to identify cancer patients at high risk for chemotherapy-induced nausea and vomiting. Ann Oncol.

[22] Navari RM, Aapro M (2016). Antiemetic prophylaxis for chemotherapy-induced nausea and vomiting. N Engl J Med.

[23] Chiu L, Chow R, Popovic M (2016). Efficacy of olanzapine for the prophylaxis and rescue of chemotherapy-induced nausea and vomiting (CINV): a systematic review and meta-analysis. Support Care Cancer.

